# Supplementary material for: TEDLH: domain HMMs for sensitive detection of remote homologues
Source: Bioinformatics. 2026 Jul 3;42(7):btag470. doi: 10.1093/bioinformatics/btag470 (PMC13385337; doi:10.1093/bioinformatics/btag470)
Supplement: btag470_Supplementary_Data [file btag470_supplementary_data.docx]

Supplementary File

TEDLH: Domain HMMs for sensitive detection of remote homologues

Claudia Alvarez Carreño^1,2^*, Anton S. Petrov^2,3^, Vaishali P. Waman^1^, Ian Sillitoe^1^, Christine Orengo^1^

^1^Department of Structural and Molecular Biology, University College London, London, United Kingdom

^2^NASA Center for the Origin of Life, Georgia Institute of Technology, Atlanta, GA 30332-0400, USA

^3^School of Chemistry and Biochemistry, Georgia Institute of Technology, 901 Atlantic Dr, Atlanta, GA 30332, USA

* Corresponding author:

Claudia Alvarez-Carreño

**Email:** c.carreno@ucl.ac.uk

## Supplementary Figure 1


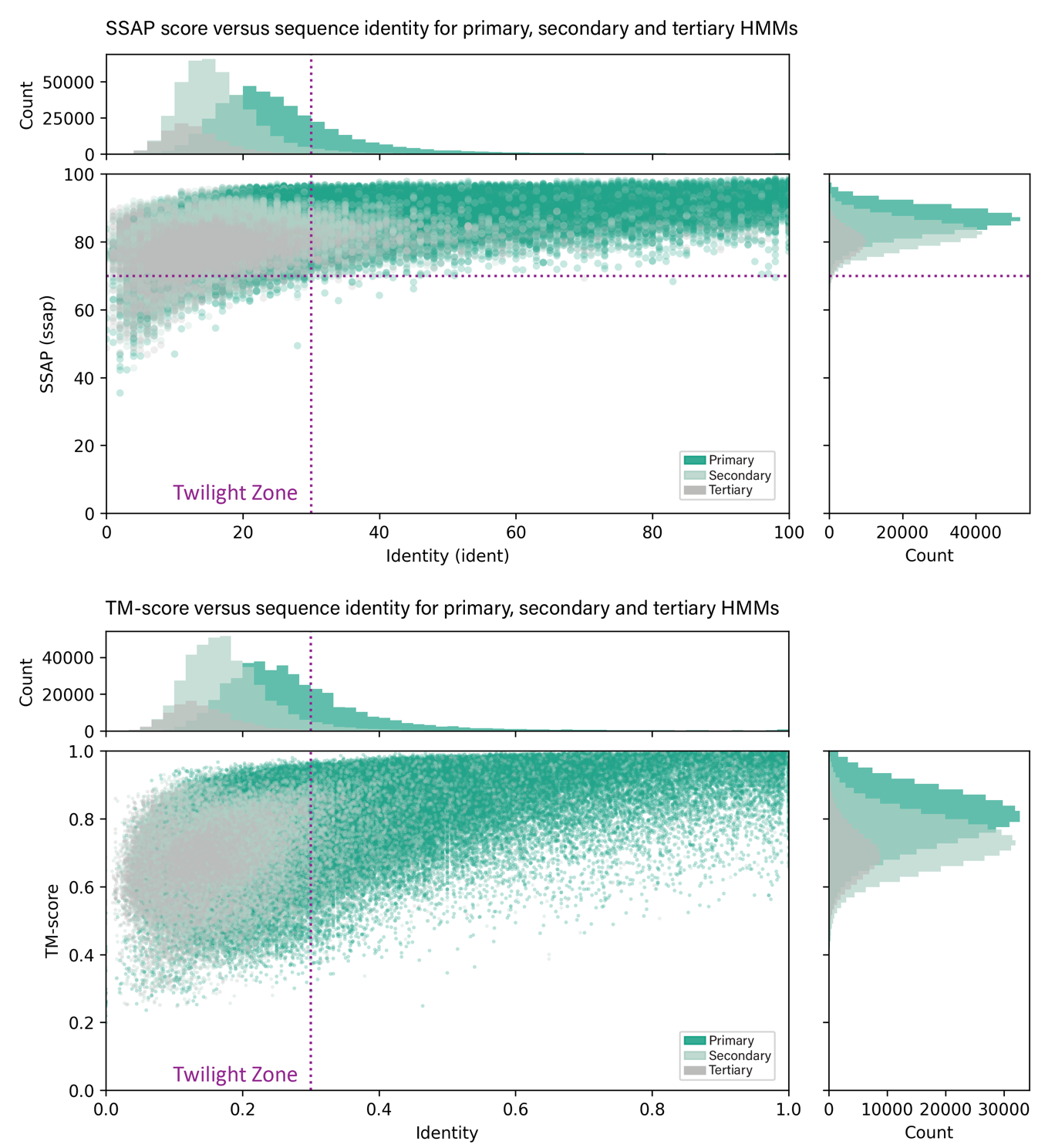


Supplementary Figure 1. Relationship between structural similarity and sequence identity for CATH-PDB structures compared with representatives of their primary, secondary, and tertiary HMMs. (a) Structure comparisons and score using the SSAP algorithm. A good structural comparison (SSAP score ≥ 70) was used as threshold for inclusion in TEDLH. (a) Structure comparisons and score using TM-align.

## Supplementary Table 2

| **CATH superfamilies not represented in TEDLH** | | | |
| --- | --- | --- | --- |
| **Superfamily** | **Domain clusters (>35% seq id):** | **Domains** | **Superfamily name** |
| 1.10.10.1030 | 1 | 3 | IrrE, HTH domain |
| 1.10.10.1120 | 1 | 1 | Lysin B, C-terminal linker domain |
| 1.10.10.1260 | 1 | 4 | Envelope glycoprotein gp160, DUF2291, helical domain |
| 1.10.10.1310 | 1 | 4 | ToxT, HTH1 motif |
| 1.10.10.1360 | 1 | 2 | tRNA (Ile)-lysidine synthase |
| 1.10.10.1460 | 1 | 1 |  |
| 1.10.10.1470 | 1 | 2 | F-112 protein-like |
| 1.10.10.1490 | 1 | 1 |  |
| 1.10.10.1660 | 1 | 4 | Nicotine adenine dinucleotide glycohydrolase, helical linker domain |
| 1.10.10.1720 | 1 | 6 | CRISPR-Cas system, Cmr2 subunit, D2 domain, helical bundle |
| 1.10.10.1740 | 2 | 3 | Transmembrane protein 14-like |
| 1.10.10.1790 | 1 | 1 |  |
| 1.10.10.180 | 2 | 57 |  |
| 1.10.10.1870 | 1 | 1 | ShTK domain-like |
| 1.10.10.190 | 2 | 2 |  |
| 1.10.10.1940 | 1 | 1 |  |
| 1.10.10.1950 | 1 | 10 |  |
| 1.10.10.1970 | 1 | 1 | TERT catalytic subunit-like |
| 1.10.10.1990 | 1 | 11 | Viral RNA-directed RNA polymerase, 4-helical domain |
| 1.10.10.2080 | 1 | 5 |  |
| 1.10.10.2170 | 1 | 11 |  |
| 1.10.10.2210 | 1 | 8 |  |
| 1.10.10.2240 | 2 | 10 |  |
| 1.10.10.2330 | 1 | 1 |  |
| 1.10.10.2380 | 2 | 7 |  |
| 1.10.10.2570 | 2 | 6 |  |
| 1.10.10.2580 | 1 | 2 | Penicillin Acylase III; Chain A, Domain 2 |
| 1.10.10.2770 | 1 | 3 |  |
| 1.10.10.2850 | 1 | 1 | Phage late-transcription coactivator-like |
| 1.10.10.2920 | 1 | 1 |  |
| 1.10.1050.20 | 1 | 1 |  |
| 1.10.10.640 | 1 | 1 | phospholipid-binding protein |
| 1.10.10.720 | 1 | 2 | leucyl-tRNA synthetase |
| 1.10.10.760 | 1 | 9 | E-set domains of sugar-utilizing enzymes |
| 1.10.1080.10 | 3 | 21 | Glutathione Synthetase; Chain A, domain 3 |
| 1.10.10.840 | 1 | 4 |  |
| 1.10.10.910 | 1 | 1 | ATP synthase, F1 beta subunit |
| 1.10.10.930 | 3 | 16 |  |
| 1.10.10.950 | 1 | 4 |  |
| 1.10.1100.10 | 1 | 1 | TAFII-230 TBP-binding domain |
| 1.10.1180.10 | 1 | 1 | B transposition protein, C-terminal domain |
| 1.10.1200.160 | 2 | 6 |  |
| 1.10.1200.170 | 1 | 4 | RNA silencing suppressor P21, C-terminal domain |
| 1.10.1200.180 | 1 | 1 |  |
| 1.10.1200.270 | 2 | 12 | Methyltransferase, alpha-helical capping domain |
| 1.10.1200.70 | 1 | 1 | Glutamyl tRNA-reductase dimerization domain |
| 1.10.1200.90 | 1 | 1 | DsbA-like domain |
| 1.10.1220.150 | 1 | 4 |  |
| 1.10.1220.60 | 1 | 2 |  |
| 1.10.1220.70 | 1 | 1 |  |
| 1.10.1220.80 | 1 | 2 |  |
| 1.10.1290.10 | 1 | 1 | Alpha trans-inducing (Alpha-TIF) |
| 1.10.1350.10 | 1 | 41 | Viral capsid alpha domain |
| 1.10.1440.10 | 1 | 3 | Apolipoprotein C-II |
| 1.10.150.160 | 1 | 1 |  |
| 1.10.150.380 | 1 | 3 | GatB domain, N-terminal subdomain |
| 1.10.150.420 | 1 | 9 | Coronavirus nonstructural protein 4 C-terminus |
| 1.10.150.460 | 1 | 3 |  |
| 1.10.150.480 | 2 | 22 |  |
| 1.10.150.540 | 1 | 3 |  |
| 1.10.150.550 | 1 | 1 | Arenavirus nucleocapsid protein, head domain |
| 1.10.150.560 | 1 | 2 |  |
| 1.10.150.610 | 1 | 1 |  |
| 1.10.150.620 | 1 | 2 | Capsid protein VP3, domain 1 |
| 1.10.150.640 | 1 | 3 | AcsD, thumb domain, helical bundle |
| 1.10.150.710 | 1 | 4 | Glutamate cysteine ligase subdomain |
| 1.10.150.740 | 1 | 5 |  |
| 1.10.150.790 | 1 | 2 |  |
| 1.10.150.830 | 1 | 2 |  |
| 1.10.150.880 | 1 | 2 |  |
| 1.10.150.90 | 6 | 34 | Immunodeficiency lentiviruses, gag gene matrix protein p17 |
| 1.10.150.920 | 1 | 4 |  |
| 1.10.168.20 | 3 | 61 | Ribosomal protein S8e, subdomain |
| 1.10.170.10 | 1 | 19 | Bluetongue Virus 10, subunit 1, domain 3 |
| 1.10.1740.120 | 1 | 2 |  |
| 1.10.1740.160 | 1 | 24 |  |
| 1.10.1740.180 | 1 | 5 |  |
| 1.10.1740.30 | 1 | 2 | Secreted effector protein SifA helical domain |
| 1.10.1740.80 | 1 | 5 |  |
| 1.10.1740.90 | 1 | 2 |  |
| 1.10.1790.50 | 1 | 1 |  |
| 1.10.1810.10 | 1 | 8 | Anti-Sigma Factor A |
| 1.10.1840.10 | 3 | 244 | main proteinase (3clpro) structure, domain 3 |
| 1.10.185.10 | 1 | 1 | Delta-retroviral matrix |
| 1.10.195.10 | 1 | 2 | HIV-1 VPU cytoplasmic domain |
| 1.10.196.20 | 1 | 2 |  |
| 1.10.196.30 | 1 | 2 |  |
| 1.10.20.120 | 1 | 1 |  |
| 1.10.2040.10 | 1 | 3 | Protein mu-1, chain B, domain 2 |
| 1.10.2050.10 | 2 | 23 | Protein mu-1, chain B, domain 3 |
| 1.10.20.70 | 1 | 1 | Transcription termination and cleavage factor, C-terminal domain |
| 1.10.210.20 | 2 | 21 |  |
| 1.10.220.120 | 2 | 6 | Sigma-70 factor, region 1.1 |
| 1.10.220.90 | 1 | 1 | Mistic |
| 1.10.238.70 | 1 | 4 |  |
| 1.10.246.100 | 1 | 1 | Vanadium-binding protein 2 |
| 1.10.246.110 | 1 | 1 | Mitochondrial ATP synthase-coupling factor 6 |
| 1.10.246.150 | 1 | 1 |  |
| 1.10.246.230 | 1 | 3 |  |
| 1.10.246.30 | 1 | 39 |  |
| 1.10.246.70 | 1 | 1 |  |
| 1.10.250.10 | 1 | 19 | Bluetongue Virus 10, subunit 1, domain 1 |
| 1.10.260.110 | 1 | 2 |  |
| 1.10.260.50 | 1 | 14 |  |
| 1.10.260.90 | 2 | 6 |  |
| 1.10.269.10 | 1 | 8 | Adenovirus DNA-binding, N-terminal domain |
| 1.10.274.110 | 1 | 2 |  |
| 1.10.274.80 | 1 | 1 |  |
| 1.10.285.20 | 1 | 4 | Uncharacterised protein PF01937, DUF89, domain 2 |
| 1.10.286.30 | 1 | 3 | Baseplate structural protein GP11, N-terminal domain |
| 1.10.286.40 | 3 | 9 | Chlorophyll a-b binding protein like |
| 1.10.287.1020 | 1 | 1 | NE0241-like |
| 1.10.287.1110 | 1 | 1 | Tubulin, GTPase |
| 1.10.287.1140 | 1 | 1 | EspA/CesA-like |
| 1.10.287.1170 | 1 | 10 | glycoside hydrolase family 81 endo-[beta] glucanase |
| 1.10.287.120 | 1 | 1 | Neurotoxin B-IV-like |
| 1.10.287.1240 | 2 | 19 |  |
| 1.10.287.1250 | 3 | 23 |  |
| 1.10.287.1350 | 2 | 31 |  |
| 1.10.287.1390 | 1 | 1 |  |
| 1.10.287.150 | 4 | 30 |  |
| 1.10.287.1520 | 2 | 6 |  |
| 1.10.287.1540 | 2 | 36 |  |
| 1.10.287.1660 | 2 | 5 |  |
| 1.10.287.170 | 1 | 2 |  |
| 1.10.287.1880 | 1 | 1 |  |
| 1.10.287.2210 | 1 | 4 |  |
| 1.10.287.2230 | 2 | 10 |  |
| 1.10.287.2480 | 3 | 12 |  |
| 1.10.287.2510 | 1 | 1 |  |
| 1.10.287.260 | 2 | 17 |  |
| 1.10.287.2720 | 1 | 11 |  |
| 1.10.287.30 | 1 | 12 | E2 (early) protein, N terminal domain, subdomain 1 |
| 1.10.287.320 | 1 | 1 | Viral phosphoprotein oligmorisation site domain |
| 1.10.287.3280 | 2 | 11 |  |
| 1.10.287.3290 | 2 | 2 |  |
| 1.10.287.340 | 1 | 1 |  |
| 1.10.287.390 | 2 | 2 |  |
| 1.10.287.3990 | 2 | 3 |  |
| 1.10.287.400 | 1 | 7 |  |
| 1.10.287.4270 | 2 | 4 |  |
| 1.10.287.490 | 1 | 2 | Helix hairpin bin |
| 1.10.287.520 | 2 | 2 | Helix hairpin bin |
| 1.10.287.570 | 1 | 1 | Helical hairpin bin |
| 1.10.287.580 | 2 | 3 | Helix hairpin bin |
| 1.10.287.700 | 2 | 2 | Helix hairpin bin |
| 1.10.287.720 | 1 | 1 | Pollen allergen ole e 6 |
| 1.10.287.790 | 1 | 8 | luxt domain from vibrio parahaemolyticus |
| 1.10.287.840 | 1 | 1 | Mycolic acid cyclopropane synthase domain like |
| 1.10.287.860 | 2 | 40 | Nucleotidyltransferase |
| 1.10.287.870 | 1 | 3 | Acyl-CoA N-acyltransferases (Nat) |
| 1.10.287.900 | 1 | 1 | The crystal structure of the spermine/spermidine acetyltransferase from enterococcus faecali |
| 1.10.287.910 | 3 | 5 | bacterial mercury transporter, merf |
| 1.10.287.920 | 1 | 1 | Pheromone alpha factor receptor. |
| 1.10.287.930 | 1 | 1 | Mammalian shaker kv1.2 potassium channel- beta subunit complex |
| 1.10.287.970 | 1 | 1 | His Kinase A (phosphoacceptor) domain |
| 1.10.3070.10 | 1 | 2 | EhaM-like |
| 1.10.3320.10 | 1 | 1 | pa2218 like domain |
| 1.10.3350.20 | 1 | 1 | Tmem141 protein family |
| 1.10.340.60 | 1 | 3 | AcsD, palm domain, helix bundle |
| 1.10.3450.20 | 1 | 4 |  |
| 1.10.3480.20 | 1 | 1 |  |
| 1.10.357.80 | 1 | 11 |  |
| 1.10.3690.10 | 1 | 8 | PA2222-like domain |
| 1.10.3730.20 | 1 | 8 |  |
| 1.10.3730.30 | 1 | 1 |  |
| 1.10.3740.10 | 1 | 2 | SSO1389-like domains |
| 1.10.3800.10 | 1 | 2 | ADP-ribosylation domain |
| 1.10.3830.10 | 1 | 3 | Diacylglycerol kinase (DAGK) domain |
| 1.10.390.40 | 1 | 7 |  |
| 1.10.3960.10 | 1 | 1 | MG354-like |
| 1.10.40.100 | 1 | 4 |  |
| 1.10.4070.10 | 1 | 2 | putative redox-enzyme maturation protein domain |
| 1.10.40.80 | 1 | 3 |  |
| 1.10.4090.10 | 1 | 93 | Viral capsid, core domain supefamily, Hepatitis B virus |
| 1.10.418.80 | 1 | 4 | Ubiquitin carboxyl-terminal hydrolase, domain 1 |
| 1.10.437.20 | 5 | 47 | dsDNA poxvirus |
| 1.10.442.10 | 1 | 95 | Cytochrome c oxidase subunit IV |
| 1.10.472.100 | 1 | 1 | Presenilin |
| 1.10.472.170 | 1 | 2 |  |
| 1.10.472.180 | 1 | 40 | Bunyavirus nucleocapsid (N) protein, C-terminal domain |
| 1.10.472.30 | 1 | 1 | Transcription elongation factor S-II, central domain |
| 1.10.490.60 | 1 | 71 | Phage p2 RNA dependent RNA polymerase domain |
| 1.10.530.50 | 1 | 6 | Peptidase U40 |
| 1.10.720.180 | 1 | 1 |  |
| 1.10.8.1010 | 1 | 1 |  |
| 1.10.8.110 | 2 | 26 | Photosystem I PsaF, reaction centre subunit III |
| 1.10.8.1130 | 1 | 2 | Bacterial toxin RNase RnlA/LsoA, C-terminal Dmd-binding domain |
| 1.10.8.1170 | 1 | 4 |  |
| 1.10.8.1190 | 2 | 38 |  |
| 1.10.8.1210 | 4 | 38 |  |
| 1.10.8.1320 | 2 | 2 |  |
| 1.10.8.1330 | 1 | 2 | Intein homing endonuclease, domain III |
| 1.10.820.10 | 2 | 70 | RNA Helicase Chain A , domain 3 |
| 1.10.8.370 | 1 | 5 | nsp7 replicase |
| 1.10.8.440 | 1 | 11 | Vesicular stomatitis virus phosphoprotein C-terminal domain |
| 1.10.8.560 | 1 | 4 | Antirestriction protein ArdA, domain 2 |
| 1.10.8.600 | 1 | 12 | Phage phi29 replication organiser protein p16.7-like |
| 1.10.8.620 | 1 | 12 | ORF12 helical bundle domain-like |
| 1.10.8.650 | 1 | 1 | Uncharacterised protein PF13642 yp_926445, C-terminal domain |
| 1.10.8.700 | 1 | 4 | Bacteriophage clamp loader A subunit, A domain |
| 1.10.8.740 | 1 | 8 |  |
| 1.10.8.790 | 1 | 7 | RNA-dependent RNA polymerase, slab domain, helical subdomain-like |
| 1.10.8.880 | 1 | 2 | Birnavirus VP3 protein, domain 2 |
| 1.10.8.890 | 1 | 1 |  |
| 1.10.890.20 | 4 | 47 |  |
| 1.10.8.960 | 1 | 4 |  |
| 1.10.8.970 | 2 | 26 | Flavivirus envelope glycoprotein M-like |
| 1.10.8.990 | 1 | 1 |  |
| 1.20.10.10 | 1 | 1 | Heat-stable enterotoxin B |
| 1.20.1050.70 | 1 | 39 | Large T antigen, SV40, domain 3 |
| 1.20.1060.20 | 1 | 4 |  |
| 1.20.1130.10 | 1 | 30 | Photosystem I PsaA/PsaB |
| 1.20.120.1010 | 1 | 1 |  |
| 1.20.120.1050 | 1 | 2 |  |
| 1.20.120.1160 | 3 | 33 |  |
| 1.20.120.1180 | 1 | 8 |  |
| 1.20.120.1260 | 1 | 6 | CRISPR-Cas system, Cmr2 subunit, D4 domain, six-helix bundle |
| 1.20.120.1350 | 3 | 55 | Pneumovirus matrix protein 2 (M2), zinc-binding domain |
| 1.20.120.1360 | 1 | 1 |  |
| 1.20.120.1390 | 1 | 11 |  |
| 1.20.120.1400 | 1 | 11 |  |
| 1.20.120.1410 | 1 | 2 |  |
| 1.20.120.1500 | 1 | 2 | Pre-hexon-linking protein IIIa |
| 1.20.120.1590 | 1 | 1 |  |
| 1.20.120.1660 | 1 | 1 |  |
| 1.20.120.1730 | 1 | 2 |  |
| 1.20.120.1850 | 1 | 5 | Ebh helix bundles repeating unit (S and A modules) |
| 1.20.120.1860 | 1 | 3 | Small t-antigen, unique domain |
| 1.20.120.1910 | 1 | 2 | Cysteine-tRNA ligase, C-terminal anti-codon recognition domain |
| 1.20.120.1980 | 1 | 2 | Knotted protein, ribbon-helix-helix DNA-binding domain |
| 1.20.120.380 | 1 | 1 | Type 1-topoisomerase catalytic fragment, domain 2 |
| 1.20.120.510 | 1 | 3 | mg296 homolog like |
| 1.20.120.620 | 1 | 1 | Backbone structure of the membrane domain of e. Coli histidine kinase receptor kdpd, |
| 1.20.120.700 | 1 | 2 | nitrate reductase, subunit delta (NarJ) |
| 1.20.120.820 | 1 | 4 | Phosphoprotein, C-terminal domain |
| 1.20.120.860 | 2 | 7 | Herpesvirus alkaline exonuclease, N-terminal domain |
| 1.20.120.950 | 1 | 1 | Uncharacterised protein DUF5062 |
| 1.20.1260.120 | 2 | 26 | Protein of unknown function DUF2935 |
| 1.20.1270.210 | 1 | 3 |  |
| 1.20.1270.230 | 1 | 6 | DNA terminal protein Gp3, priming domain |
| 1.20.1270.270 | 1 | 24 | VP1, C-terminal extension domain |
| 1.20.1270.310 | 1 | 7 |  |
| 1.20.1270.320 | 1 | 9 | Poxvirus poly(A) polymerase, N domain |
| 1.20.1270.410 | 1 | 2 |  |
| 1.20.1270.460 | 1 | 4 |  |
| 1.20.1280.120 | 1 | 3 |  |
| 1.20.1280.150 | 1 | 12 | Hepatitis C virus non-structural protein NS2, N-terminal domain |
| 1.20.1280.160 | 1 | 8 |  |
| 1.20.1280.200 | 1 | 5 | Orbivirus VP4 core protein, C-terminal domain |
| 1.20.1280.210 | 1 | 1 |  |
| 1.20.1280.260 | 1 | 26 |  |
| 1.20.1280.310 | 1 | 3 |  |
| 1.20.1400.10 | 1 | 2 | Attractin |
| 1.20.140.110 | 1 | 23 |  |
| 1.20.140.190 | 1 | 17 |  |
| 1.20.142.20 | 1 | 40 |  |
| 1.20.1440.190 | 1 | 4 | Tenuivirus movement protein |
| 1.20.1440.300 | 1 | 2 | RNA-directed RNA polymerase L, helical domain |
| 1.20.1440.330 | 1 | 1 |  |
| 1.20.1440.360 | 1 | 4 |  |
| 1.20.1440.370 | 1 | 2 |  |
| 1.20.1480.10 | 1 | 1 | hypothetical protein mp506/mpn330, domain 1 |
| 1.20.1480.30 | 2 | 4 | Designed four-helix bundle protein |
| 1.20.1570.10 | 1 | 4 | dip2346 domain like |
| 1.20.1690.20 | 2 | 8 |  |
| 1.20.1700.20 | 1 | 3 |  |
| 1.20.190.40 | 1 | 2 | Viral ssDNA binding protein, head domain |
| 1.20.272.30 | 1 | 2 |  |
| 1.20.272.50 | 1 | 4 | Bacteriophage clamp loader A subunit, A' domain |
| 1.20.272.60 | 1 | 2 |  |
| 1.20.50.10 | 1 | 3 | Pheromone ER-1 |
| 1.20.50.50 | 1 | 1 |  |
| 1.20.50.60 | 1 | 1 | ESP1, core domain |
| 1.20.50.70 | 1 | 2 |  |
| 1.20.5.10 | 2 | 37 |  |
| 1.20.5.100 | 11 | 232 | Cytochrome c1, transmembrane anchor, C-terminal |
| 1.20.5.1000 | 1 | 2 | arf6 gtpase in complex with a specific effector, jip4 |
| 1.20.5.1010 | 1 | 8 | TRPM, tetramerisation domain |
| 1.20.5.1030 | 1 | 1 | Preprotein translocase secy subunit |
| 1.20.5.1040 | 1 | 1 | Sensor protein qsec. |
| 1.20.5.1060 | 1 | 24 | Tetrabrachion |
| 1.20.5.1070 | 1 | 2 | Head and neck region of the ectodomain of NDV fusion glycoprotein |
| 1.20.5.1130 | 1 | 1 | Connexin43 |
| 1.20.5.1140 | 1 | 2 | Docking domain of the erythromycin polyketide synthase (DEBS) |
| 1.20.5.1150 | 1 | 70 | Ribosomal protein S8 |
| 1.20.5.1160 | 5 | 13 | Vasodilator-stimulated phosphoprotein |
| 1.20.5.1170 | 1 | 4 | HIT-like |
| 1.20.5.1180 | 5 | 46 | Geminin coiled-coil domain |
| 1.20.5.1190 | 3 | 12 | iswi atpase |
| 1.20.5.120 | 1 | 7 | Proteasome activator pa28, N-terminal domain |
| 1.20.5.1200 | 1 | 10 | Alpha-tocopherol transfer |
| 1.20.5.1210 | 1 | 4 | Integron cassette protein helical domain |
| 1.20.5.1220 | 1 | 2 | Regulatory protein rop |
| 1.20.5.1230 | 1 | 4 | Apolipoprotein A-I |
| 1.20.5.1240 | 1 | 2 | Endo-n-acetylneuraminidase |
| 1.20.5.130 | 2 | 4 |  |
| 1.20.5.1350 | 1 | 10 |  |
| 1.20.5.140 | 2 | 3 |  |
| 1.20.5.1450 | 3 | 5 |  |
| 1.20.5.1500 | 4 | 13 |  |
| 1.20.5.160 | 1 | 7 | Bacterial aa3 type cytochrome c oxidase subunit IV |
| 1.20.5.1610 | 2 | 14 |  |
| 1.20.5.1700 | 7 | 30 |  |
| 1.20.5.1760 | 2 | 5 |  |
| 1.20.5.1890 | 2 | 28 |  |
| 1.20.5.190 | 5 | 25 |  |
| 1.20.5.20 | 1 | 3 |  |
| 1.20.5.2120 | 1 | 1 |  |
| 1.20.5.220 | 12 | 38 |  |
| 1.20.5.2210 | 2 | 3 |  |
| 1.20.5.2230 | 2 | 6 |  |
| 1.20.5.2280 | 2 | 22 |  |
| 1.20.5.230 | 1 | 16 |  |
| 1.20.5.240 | 1 | 8 |  |
| 1.20.5.2480 | 2 | 3 |  |
| 1.20.5.2620 | 2 | 2 |  |
| 1.20.5.2710 | 2 | 3 |  |
| 1.20.5.290 | 1 | 24 | Phospholamban |
| 1.20.5.2950 | 2 | 7 |  |
| 1.20.5.30 | 1 | 3 |  |
| 1.20.5.300 | 13 | 56 |  |
| 1.20.5.3070 | 3 | 21 |  |
| 1.20.5.360 | 1 | 3 | SFTPD helical domain |
| 1.20.5.3600 | 2 | 3 |  |
| 1.20.5.370 | 6 | 39 |  |
| 1.20.5.3730 | 2 | 14 |  |
| 1.20.5.3790 | 2 | 28 |  |
| 1.20.5.3800 | 2 | 6 |  |
| 1.20.5.390 | 6 | 23 | L1 transposable element, trimerization domain |
| 1.20.5.3960 | 2 | 3 |  |
| 1.20.5.400 | 1 | 14 |  |
| 1.20.5.4090 | 2 | 5 |  |
| 1.20.5.430 | 5 | 54 |  |
| 1.20.5.450 | 1 | 1 |  |
| 1.20.5.4520 | 1 | 6 |  |
| 1.20.5.4570 | 2 | 24 |  |
| 1.20.5.460 | 12 | 28 | Single helix bin |
| 1.20.5.4730 | 4 | 8 |  |
| 1.20.5.4770 | 2 | 12 |  |
| 1.20.5.4880 | 2 | 19 |  |
| 1.20.5.490 | 6 | 33 | Single helix bin |
| 1.20.5.4980 | 2 | 29 |  |
| 1.20.5.500 | 5 | 11 | Single helix bin |
| 1.20.5.5100 | 2 | 4 |  |
| 1.20.5.5160 | 3 | 18 |  |
| 1.20.5.520 | 1 | 1 | Single helix bin |
| 1.20.5.5200 | 3 | 70 |  |
| 1.20.5.5270 | 1 | 6 |  |
| 1.20.5.530 | 1 | 2 | Single helix bin |
| 1.20.5.540 | 4 | 55 | Single helix bin |
| 1.20.5.550 | 1 | 1 | Single Helix bin |
| 1.20.5.560 | 1 | 64 | Single Heli x bin |
| 1.20.5.570 | 2 | 6 | Single helix bin |
| 1.20.5.580 | 3 | 11 | Single Helix bin |
| 1.20.5.590 | 1 | 1 | Single helix bin |
| 1.20.5.600 | 1 | 1 | Potassium channel, voltage dependent, Kv1.4, tandem inactivation domain |
| 1.20.5.620 | 5 | 8 | F1F0 ATP synthase subunit B, membrane domain |
| 1.20.5.630 | 1 | 1 | Integrin beta subunit, cytoplasmic domain |
| 1.20.5.640 | 6 | 10 | Single helix bin |
| 1.20.5.650 | 2 | 4 | Single helix bin |
| 1.20.5.680 | 1 | 10 | Single Helix bin |
| 1.20.5.70 | 2 | 10 |  |
| 1.20.5.710 | 2 | 9 | Single helix bin |
| 1.20.5.740 | 5 | 9 | Single helix bin |
| 1.20.5.750 | 1 | 3 | Moricin domain |
| 1.20.5.760 | 1 | 1 | Single helix bin |
| 1.20.5.770 | 1 | 1 | Single helix bin |
| 1.20.5.790 | 3 | 8 | Single helix bin |
| 1.20.5.80 | 2 | 77 |  |
| 1.20.5.800 | 1 | 2 | Alr1493-like domains |
| 1.20.58.1200 | 1 | 4 | RNA silencing suppressor P21, N-terminal domain |
| 1.20.58.1230 | 1 | 3 | Rac1-binding domain, C-terminal subdomain |
| 1.20.58.130 | 1 | 2 |  |
| 1.20.58.1340 | 1 | 1 |  |
| 1.20.58.1510 | 1 | 1 |  |
| 1.20.58.1560 | 2 | 4 |  |
| 1.20.58.1650 | 1 | 2 |  |
| 1.20.58.1680 | 1 | 12 |  |
| 1.20.58.1860 | 1 | 3 |  |
| 1.20.58.1880 | 1 | 46 |  |
| 1.20.58.1950 | 1 | 4 |  |
| 1.20.58.2030 | 1 | 6 |  |
| 1.20.58.2070 | 1 | 2 |  |
| 1.20.58.2170 | 1 | 9 |  |
| 1.20.58.2180 | 1 | 4 |  |
| 1.20.58.2230 | 1 | 2 | Retrograde transport protein Dsl1, N-terminal domain |
| 1.20.5.830 | 1 | 8 | luxt domain from vibrio parahaemolyticus |
| 1.20.58.350 | 1 | 4 | Thioesterase/thiol ester dehydrase-isomerase |
| 1.20.5.840 | 1 | 2 | hypothetical RNA methyltransferase |
| 1.20.5.850 | 1 | 1 | Rbstp2229 protein |
| 1.20.58.520 | 1 | 1 | Amidohydrolase |
| 1.20.58.540 | 1 | 3 |  |
| 1.20.58.620 | 1 | 1 |  |
| 1.20.5.870 | 1 | 106 | Voltage-gated potassium channel |
| 1.20.58.730 | 1 | 1 |  |
| 1.20.58.770 | 1 | 1 |  |
| 1.20.58.780 | 1 | 1 |  |
| 1.20.58.850 | 1 | 1 |  |
| 1.20.58.940 | 1 | 2 |  |
| 1.20.58.960 | 1 | 1 | Protein of unknown function (DUF3120) |
| 1.20.5.90 | 1 | 3 | VpR/VpX protein, C-terminal domain |
| 1.20.5.900 | 1 | 1 | transmembrane domain of human cd4 |
| 1.20.5.920 | 1 | 3 | rhodobacter sphaeroides pufx membrane protein |
| 1.20.5.930 | 2 | 4 | Bicelle-embedded integrin alpha(iib) transmembrane segment |
| 1.20.5.950 | 1 | 1 | bacteriochlorophyll c-binding protein |
| 1.20.5.960 | 1 | 4 | Bacteriophage t4 gene product 9 (gp9) |
| 1.20.5.970 | 1 | 2 | Nonstructural RNA-binding protein |
| 1.20.5.990 | 3 | 18 | Nemo cc2-lz domain - 1d5 darpin complex |
| 1.20.860.20 | 1 | 2 | Photosystem I PsaK, reaction centre |
| 1.20.890.100 | 1 | 4 |  |
| 1.20.890.20 | 2 | 31 | mpn423 like domain |
| 1.20.890.30 | 1 | 1 | VCA0319-like |
| 1.20.890.70 | 1 | 3 | Protein translocase subunit SecA, preprotein binding domain |
| 1.20.89.10 | 1 | 95 | Nitrogenase Molybdenum-iron Protein, subunit B, domain 4 |
| 1.20.89.20 | 2 | 13 |  |
| 1.20.91.10 | 1 | 57 |  |
| 1.20.920.50 | 2 | 8 |  |
| 1.20.920.70 | 1 | 6 |  |
| 1.20.960.20 | 7 | 227 |  |
| 1.20.970.20 | 1 | 1 | Glycogen synthesis protein GlgS |
| 1.25.10.100 | 1 | 2 |  |
| 1.25.10.50 | 1 | 12 |  |
| 1.25.10.60 | 1 | 3 | Rad61, Wapl domain |
| 1.25.10.70 | 1 | 1 |  |
| 1.25.20.10 | 1 | 3 | Bacterial muramidases |
| 1.25.40.1010 | 1 | 8 |  |
| 1.25.40.1030 | 1 | 4 |  |
| 1.25.40.120 | 4 | 153 | Protein prenylyltransferase |
| 1.25.40.300 | 2 | 13 | Putative secreted effector protein |
| 1.25.40.350 | 1 | 2 |  |
| 1.25.40.440 | 1 | 2 | Nucleoporin, helical domain, central subdomain |
| 1.25.40.460 | 1 | 6 |  |
| 1.25.40.620 | 1 | 5 |  |
| 1.25.40.640 | 1 | 1 | Avirulence protein ATR13 |
| 1.25.40.670 | 1 | 1 |  |
| 1.25.40.730 | 1 | 16 |  |
| 1.25.40.830 | 1 | 12 |  |
| 1.25.40.860 | 1 | 1 |  |
| 2.10.10.40 | 1 | 1 |  |
| 2.10.10.70 | 1 | 65 | Filoviridae VP35, C-terminal inhibitory domain, beta-sheet subdomain |
| 2.10.110.20 | 1 | 43 |  |
| 2.10.210.10 | 3 | 50 | Cytochrome Bc1 Complex; Chain I |
| 2.10.25.160 | 1 | 2 | Granulin |
| 2.10.25.20 | 6 | 32 | reovirus attachment protein sigma1; domain 1 |
| 2.10.25.60 | 1 | 2 |  |
| 2.10.280.10 | 1 | 1 | heat- and protease-stable fragment of the bacteriophage t4 short fibre, domain 1 |
| 2.10.310.10 | 1 | 2 | Serpins superfamily |
| 2.10.50.20 | 1 | 1 | Inosine monophosphate dehydrogenase (IMPDH) |
| 2.10.50.40 | 1 | 4 |  |
| 2.105.10.10 | 1 | 1 | Pseudo beta propeller |
| 2.10.70.30 | 1 | 1 | Ta1353-like |
| 2.10.70.50 | 1 | 3 |  |
| 2.10.70.60 | 3 | 3 | Phospholipase B-like, domain 1 |
| 2.10.77.10 | 9 | 795 | Hemagglutinin Chain A, Domain 2 |
| 2.115.10.10 | 1 | 2 | Tachylectin 2 |
| 2.150.10.20 | 1 | 2 |  |
| 2.160.20.130 | 1 | 15 |  |
| 2.160.20.160 | 1 | 4 |  |
| 2.160.20.180 | 1 | 2 |  |
| 2.160.20.20 | 7 | 39 |  |
| 2.160.30.10 | 1 | 1 | heat- and protease-stable fragment of the bacteriophage t4 short fibre, domain 2 |
| 2.170.160.10 | 1 | 39 | Endo-1,4-beta-glucanase f. Domain 2 |
| 2.170.200.10 | 3 | 13 | Papillomavirus E2 early protein domain |
| 2.170.290.10 | 1 | 14 | baseplate structural protein gp8, domain 2 |
| 2.170.30.10 | 4 | 171 | Parvovirus coat protein VP1/VP2 |
| 2.170.40.20 | 4 | 195 | Human immunodeficiency virus 1, Gp160, envelope glycoprotein |
| 2.170.9.10 | 1 | 19 | Adenovirus Type 2 Hexon, domain 1 |
| 2.20.140.20 | 1 | 8 |  |
| 2.20.150.10 | 4 | 11 | putative 5-dehydro-2- deoxygluconokinase |
| 2.20.170.10 | 1 | 2 | p120GAP domain-like |
| 2.20.20.130 | 3 | 5 |  |
| 2.20.20.20 | 1 | 3 | Baseplate structural protein gp11, C-terminal domain |
| 2.20.20.30 | 2 | 3 | reverse gyrase domain |
| 2.20.20.40 | 1 | 4 | Integron cassette protein |
| 2.20.20.60 | 1 | 1 |  |
| 2.20.210.30 | 1 | 27 |  |
| 2.20.230.30 | 1 | 4 |  |
| 2.20.25.120 | 1 | 4 |  |
| 2.20.25.130 | 1 | 3 | Zinc stem-like domain |
| 2.20.25.140 | 1 | 6 |  |
| 2.20.25.160 | 1 | 24 |  |
| 2.20.25.180 | 1 | 1 |  |
| 2.20.25.210 | 1 | 10 | Hepatitis C NS5A, domain 1B |
| 2.20.25.220 | 1 | 10 | Hepatitis C virus NS5A, 1B domain |
| 2.20.25.230 | 1 | 1 |  |
| 2.20.25.340 | 1 | 36 |  |
| 2.20.25.370 | 1 | 1 | Ipi1, C-terminal domain |
| 2.20.25.400 | 2 | 5 |  |
| 2.20.25.440 | 1 | 2 |  |
| 2.20.25.450 | 1 | 3 |  |
| 2.20.25.500 | 1 | 8 |  |
| 2.20.25.510 | 1 | 1 |  |
| 2.20.25.540 | 1 | 1 |  |
| 2.20.25.560 | 1 | 1 |  |
| 2.20.28.150 | 1 | 1 |  |
| 2.20.28.180 | 1 | 1 | Arenavirus glycoprotein, zinc binding domain |
| 2.20.28.300 | 1 | 2 |  |
| 2.20.28.50 | 4 | 9 | degv family protein |
| 2.20.60.10 | 1 | 2 | Pleiotrophin/Midkine, N-terminal domain |
| 2.20.70.150 | 1 | 1 |  |
| 2.20.70.20 | 1 | 12 |  |
| 2.20.70.60 | 1 | 2 |  |
| 2.20.70.70 | 2 | 3 |  |
| 2.30.130.120 | 1 | 4 |  |
| 2.30.140.40 | 1 | 9 | Pestivirus Npro endopeptidase C53, interaction domain |
| 2.30.26.10 | 1 | 182 | Dihydroorotate Dehydrogenase A, chain A, domain 2 |
| 2.30.270.20 | 1 | 69 |  |
| 2.30.29.100 | 5 | 34 |  |
| 2.30.29.130 | 2 | 11 |  |
| 2.30.30.1000 | 1 | 2 | Replicase polyprotein 1a |
| 2.30.30.1150 | 1 | 4 |  |
| 2.30.30.1240 | 1 | 3 | AscD, thumb domain, four stranded beta-sheet |
| 2.30.30.1250 | 2 | 18 |  |
| 2.30.30.420 | 1 | 2 | glucansucrase |
| 2.30.30.470 | 1 | 2 | Penicillin-binding protein Tp47, domain B |
| 2.30.30.480 | 1 | 1 |  |
| 2.30.30.600 | 1 | 1 |  |
| 2.30.30.620 | 1 | 16 |  |
| 2.30.30.630 | 1 | 1 |  |
| 2.30.30.640 | 1 | 11 |  |
| 2.30.30.650 | 1 | 4 |  |
| 2.30.30.660 | 1 | 5 | Protein of unknown function (DUF3539) |
| 2.30.30.710 | 1 | 12 | Hepatitis C virus non-structural protein NS2, C-terminal domain |
| 2.30.30.780 | 1 | 3 |  |
| 2.30.30.810 | 1 | 3 |  |
| 2.30.30.880 | 1 | 4 |  |
| 2.30.30.980 | 1 | 1 |  |
| 2.30.31.30 | 1 | 1 | Arterivirus nps1beta, nuclease domain |
| 2.30.340.10 | 1 | 6 | PAZ domain superfamily |
| 2.30.36.90 | 1 | 1 |  |
| 2.30.42.30 | 1 | 2 |  |
| 2.30.90.10 | 1 | 1 | Heparin-binding Growth Factor, Midkine; Chain A- C-terminal Domain |
| 2.40.10.200 | 1 | 1 | STY4665 C-terminal domain-like |
| 2.40.10.250 | 2 | 16 | Replicase NSP9 |
| 2.40.10.380 | 1 | 5 |  |
| 2.40.10.510 | 1 | 3 |  |
| 2.40.160.220 | 1 | 6 |  |
| 2.40.30.120 | 7 | 322 | Positive stranded ssRNA viruses |
| 2.40.30.150 | 1 | 3 | Bacteriophage T4, Gp27, baseplate hub, domain 3 |
| 2.40.30.190 | 1 | 3 |  |
| 2.40.30.250 | 1 | 33 |  |
| 2.40.37.30 | 1 | 1 |  |
| 2.40.490.10 | 4 | 20 | Newcastle disease virus like domain |
| 2.40.50.300 | 1 | 2 |  |
| 2.40.50.420 | 1 | 4 | Envelope glycoprotein gp160, DUF2291, alpha/beta domain |
| 2.40.50.470 | 1 | 2 |  |
| 2.40.50.510 | 1 | 3 |  |
| 2.40.50.610 | 1 | 4 | Type II restriction enzyme SfiI, DNA-recognition domain |
| 2.40.50.710 | 1 | 1 |  |
| 2.40.50.830 | 1 | 6 |  |
| 2.40.50.930 | 1 | 62 |  |
| 2.40.510.10 | 2 | 322 | Positive stranded ssRNA viruses |
| 2.60.120.1010 | 2 | 10 |  |
| 2.60.120.1100 | 1 | 1 |  |
| 2.60.120.1150 | 1 | 1 |  |
| 2.60.120.1240 | 1 | 6 |  |
| 2.60.120.1300 | 1 | 1 |  |
| 2.60.120.1310 | 1 | 20 |  |
| 2.60.120.1320 | 1 | 20 |  |
| 2.60.120.1330 | 1 | 20 |  |
| 2.60.120.1400 | 1 | 11 | Viral head domain of polysaccharide receptor-binding protein-like |
| 2.60.120.1450 | 1 | 4 |  |
| 2.60.120.1680 | 2 | 38 |  |
| 2.60.120.170 | 4 | 76 |  |
| 2.60.120.220 | 3 | 86 | Satellite virus coat domain |
| 2.60.120.420 | 2 | 23 | Membrane penetration protein mu1, Chain B, domain 4 |
| 2.60.120.530 | 1 | 6 | Cucumovirus coat protein, subunit A |
| 2.60.120.550 | 1 | 26 | Penton protein; domain 1 |
| 2.60.120.640 | 1 | 4 | gp9 |
| 2.60.120.660 | 2 | 47 | icosahedral virus |
| 2.60.120.670 | 1 | 6 | Minor capsid protein. |
| 2.60.120.730 | 2 | 12 |  |
| 2.60.120.800 | 1 | 27 | Rotavirus outer-layer protein VP7, domain 2 |
| 2.60.120.950 | 2 | 82 | Circovirus capsid protein |
| 2.60.120.960 | 1 | 5 | Spike glycoprotein, N-terminal domain |
| 2.60.169.10 | 1 | 6 | Microviridae F protein |
| 2.60.175.10 | 4 | 409 | Capsid protein VP1,Polyomavirus |
| 2.60.175.20 | 1 | 141 | Major capsid L1 (late) superfamily, Papillomavirus |
| 2.60.240.10 | 2 | 7 | Major secreted virus protein |
| 2.60.240.20 | 1 | 3 |  |
| 2.60.240.30 | 1 | 4 |  |
| 2.60.250.10 | 1 | 10 | Baculovirus p35 |
| 2.60.260.50 | 1 | 2 | Flavivirus polyprotein propeptide domain |
| 2.60.270.70 | 2 | 8 |  |
| 2.60.320.20 | 1 | 2 | Pestivirus envelope glycoprotein E2, domain A |
| 2.60.330.10 | 1 | 1 | receptor-binding protein prd1-p2, domain 2 |
| 2.60.40.1200 | 1 | 4 |  |
| 2.60.40.1330 | 1 | 8 |  |
| 2.60.40.1340 | 1 | 8 | Chemokine-binding protein M3-like |
| 2.60.40.1550 | 1 | 2 | SARS coronavirus X4 |
| 2.60.40.1680 | 1 | 4 | 4-oxalocrotonate tautomerase-like |
| 2.60.40.1690 | 3 | 20 | Head and neck region of the ectodomain of NDV fusion glycoprotein |
| 2.60.40.1780 | 1 | 3 | Carmovirus coat protein |
| 2.60.40.1900 | 1 | 1 | Beta-microseminoprotein (PSP94) domain |
| 2.60.40.1960 | 1 | 8 |  |
| 2.60.40.1990 | 1 | 2 |  |
| 2.60.40.2400 | 1 | 14 | Alphavirus E2 glycoprotein, domain C |
| 2.60.40.2530 | 1 | 2 |  |
| 2.60.40.2650 | 1 | 12 | Rubella membrane glycoprotein E1, domain 3 |
| 2.60.40.2760 | 1 | 10 |  |
| 2.60.40.2770 | 2 | 13 | WSSV envelope protein-like |
| 2.60.40.2800 | 1 | 1 |  |
| 2.60.40.2810 | 1 | 1 |  |
| 2.60.40.2820 | 1 | 1 |  |
| 2.60.40.2900 | 1 | 3 |  |
| 2.60.40.2920 | 2 | 4 |  |
| 2.60.40.2930 | 1 | 7 |  |
| 2.60.40.2940 | 1 | 10 |  |
| 2.60.40.3000 | 1 | 6 | Pestivirus envelope glycoprotein E2, domain B |
| 2.60.40.3040 | 1 | 2 |  |
| 2.60.40.3130 | 2 | 8 |  |
| 2.60.40.3190 | 2 | 3 | Herpesvirus glycoprotein H, C-terminal domain |
| 2.60.40.3200 | 2 | 14 | Alphavirus E2 glycoprotein, A domain |
| 2.60.40.3420 | 1 | 1 |  |
| 2.60.40.3460 | 1 | 2 |  |
| 2.60.40.350 | 4 | 209 |  |
| 2.60.40.3530 | 1 | 8 |  |
| 2.60.40.3580 | 1 | 6 |  |
| 2.60.40.3790 | 1 | 4 |  |
| 2.60.40.4030 | 2 | 8 |  |
| 2.60.40.4200 | 1 | 2 | Pestivirus envelope glycoprotein E2, C-terminal domain |
| 2.60.40.4260 | 1 | 4 |  |
| 2.60.40.4280 | 1 | 2 |  |
| 2.60.40.4310 | 1 | 8 | Alphavirus E2 glycoprotein, domain B |
| 2.60.40.4340 | 1 | 2 |  |
| 2.60.40.4360 | 1 | 1 |  |
| 2.60.40.4370 | 1 | 1 |  |
| 2.60.430.10 | 1 | 1 | YopX-like domain |
| 2.60.500.10 | 1 | 2 | Surface Active Protein domain |
| 2.60.510.10 | 1 | 8 | EV matrix protein |
| 2.60.90.10 | 5 | 198 | Adenovirus pIV-related, attachment domain |
| 2.60.90.20 | 2 | 46 | Virus attachment protein , globular domain |
| 2.60.90.30 | 2 | 15 | Fiber protein 1, C-terminal domain |
| 2.60.90.40 | 1 | 7 |  |
| 2.60.90.50 | 1 | 5 |  |
| 2.60.98.10 | 4 | 120 | Tick-borne Encephalitis virus Glycoprotein, domain 1 |
| 2.60.98.30 | 1 | 12 | Rubella membrane glycoprotein E1, domain 1 |
| 2.60.98.50 | 1 | 4 |  |
| 2.70.10.10 | 3 | 13 | Thrombin Inhibitor (Hirudin), subunit I |
| 2.70.20.20 | 2 | 16 | Matrix protein VP40, N-terminal domain |
| 2.70.20.30 | 1 | 8 | HRSV-S2 matrix protein, N-terminal domain |
| 2.70.20.40 | 1 | 13 | Borna disease virus, matrix protein |
| 2.70.20.50 | 1 | 6 | Viral matrix protein, N-terminal domain |
| 2.70.20.60 | 1 | 6 | Viral matrix protein, C-terminal domain |
| 2.70.230.10 | 4 | 18 |  |
| 2.70.240.20 | 1 | 1 | Leukocidin/Hemolysin toxin, cytolysin domain |
| 2.70.250.10 | 1 | 2 | receptor-binding protein prd1-p2, domain 3 |
| 2.70.40.20 | 1 | 1 | Baculovirus telokin-like protein 20 |
| 2.70.9.30 | 1 | 9 | Viral coat protein p3 |
| 2.70.98.100 | 1 | 2 | Baculovirus E66 occlusion-derived virus envelope protein, domain 2 |
| 2.90.10.30 | 1 | 6 |  |
| 3.10.10.20 | 1 | 8 |  |
| 3.10.20.260 | 1 | 28 | NEDD8-activating enzyme E1, catalytic subunit |
| 3.10.20.330 | 1 | 2 | Protein of unknown function YopT |
| 3.10.20.350 | 1 | 3 |  |
| 3.10.20.540 | 2 | 32 |  |
| 3.10.20.750 | 1 | 2 |  |
| 3.10.20.770 | 2 | 12 |  |
| 3.10.20.820 | 1 | 14 |  |
| 3.10.360.40 | 1 | 1 |  |
| 3.10.390.20 | 1 | 19 | Viral glycoprotein L |
| 3.10.450.130 | 1 | 1 | folded 79 residue fragment of lin0334 like domains |
| 3.10.450.20 | 1 | 41 | Bacteriophage PBS2, uracil-glycosylase inhibitor |
| 3.10.450.210 | 1 | 13 |  |
| 3.10.450.260 | 1 | 1 |  |
| 3.10.450.380 | 1 | 2 |  |
| 3.10.450.630 | 1 | 2 |  |
| 3.10.450.730 | 1 | 7 | BLIP domain |
| 3.10.460.10 | 1 | 3 | VSV matrix protein |
| 3.10.460.20 | 1 | 1 | Rhabdovirus matrix protein M2 |
| 3.10.470.10 | 1 | 4 | Chromosomal protein MC1 |
| 3.10.50.50 | 1 | 13 | Rubella virus capsid protein |
| 3.10.50.90 | 1 | 3 |  |
| 3.10.570.10 | 2 | 6 | sex pheromone staph- cam373 precursor domain |
| 3.20.100.20 | 1 | 6 |  |
| 3.20.16.10 | 3 | 73 | Herpesvirus/Caudovirus protease domain |
| 3.30.10.30 | 1 | 3 | DYRK |
| 3.30.1040.10 | 1 | 8 | Carboxypeptidase inhibitor |
| 3.30.1040.20 | 3 | 15 |  |
| 3.30.1040.70 | 1 | 4 |  |
| 3.30.1070.20 | 1 | 2 |  |
| 3.30.110.130 | 1 | 1 | Hemolytic toxin, N-terminal domain |
| 3.30.110.140 | 1 | 1 |  |
| 3.30.110.180 | 1 | 2 |  |
| 3.30.1110.10 | 2 | 47 |  |
| 3.30.1170.10 | 1 | 4 |  |
| 3.30.1190.10 | 1 | 1 | DNA-binding protein Tfx superfamily, archaea |
| 3.30.1300.70 | 1 | 5 |  |
| 3.30.1300.90 | 1 | 2 | PilM protein, N-terminal domain |
| 3.30.1310.30 | 1 | 1 |  |
| 3.30.1330.220 | 1 | 1 | Arterivirus nonstructural protein 7 alpha |
| 3.30.1330.230 | 1 | 1 |  |
| 3.30.1350.20 | 1 | 39 | Bacteriophage PHI-29 conector. Domain 3 |
| 3.30.1360.240 | 1 | 4 |  |
| 3.30.1370.130 | 1 | 2 |  |
| 3.30.1370.190 | 1 | 2 |  |
| 3.30.1370.200 | 1 | 2 |  |
| 3.30.1390.40 | 1 | 26 | Ribosomal protein L30p/L7e |
| 3.30.1490.160 | 1 | 1 | ctc02137 like domains |
| 3.30.1490.200 | 1 | 2 | Penicillin-binding protein Tp47, domain A |
| 3.30.1490.250 | 1 | 4 |  |
| 3.30.1490.260 | 1 | 1 |  |
| 3.30.1490.430 | 1 | 2 |  |
| 3.30.160.120 | 1 | 2 | Hypothetical protein MTH1880 |
| 3.30.160.290 | 1 | 15 | Rag2 PHD finger |
| 3.30.160.300 | 2 | 7 |  |
| 3.30.160.310 | 1 | 2 |  |
| 3.30.160.400 | 1 | 1 |  |
| 3.30.160.490 | 1 | 1 |  |
| 3.30.160.510 | 1 | 1 | Histone-like nucleoid-structuring protein H-NS |
| 3.30.160.540 | 1 | 1 |  |
| 3.30.160.560 | 1 | 2 |  |
| 3.30.160.590 | 1 | 1 |  |
| 3.30.160.710 | 1 | 1 |  |
| 3.30.160.80 | 1 | 3 | Amb V Allergen |
| 3.30.160.820 | 1 | 23 | Nsp15 N-terminal domain-like |
| 3.30.160.850 | 1 | 1 |  |
| 3.30.160.860 | 1 | 1 |  |
| 3.30.160.870 | 1 | 3 |  |
| 3.30.160.890 | 1 | 1 | Hepatitis C virus envelope glycoprotein E1, chain C |
| 3.30.1680.40 | 1 | 1 |  |
| 3.30.1710.10 | 3 | 7 | top7, de novo designed protein |
| 3.30.1900.20 | 1 | 3 |  |
| 3.30.1920.40 | 1 | 3 |  |
| 3.30.1920.50 | 1 | 133 |  |
| 3.30.200.200 | 1 | 13 |  |
| 3.30.200.210 | 1 | 12 |  |
| 3.30.200.260 | 1 | 2 |  |
| 3.30.200.70 | 1 | 1 |  |
| 3.30.200.90 | 2 | 2 |  |
| 3.30.2020.50 | 1 | 16 |  |
| 3.30.2200.10 | 1 | 2 | histidine kinase doma clostridium symbiosum atcc 14940 |
| 3.30.2240.10 | 1 | 16 | mRNA decapping enzyme DcpS N-terminal domain |
| 3.30.2250.10 | 2 | 4 | Bifunctional DNA primase/polymerase domain |
| 3.30.230.110 | 2 | 28 |  |
| 3.30.230.140 | 1 | 11 |  |
| 3.30.2320.40 | 1 | 1 |  |
| 3.30.2330.20 | 1 | 19 | family 98 glycoside hydrolase |
| 3.30.240.30 | 1 | 2 |  |
| 3.30.240.40 | 3 | 14 | E6 early regulatory protein |
| 3.30.2460.10 | 1 | 2 | Endo-n-acetylneuraminidase domain |
| 3.30.300.320 | 1 | 11 |  |
| 3.30.30.100 | 2 | 2 |  |
| 3.30.30.130 | 1 | 2 |  |
| 3.30.30.140 | 1 | 1 |  |
| 3.30.30.180 | 1 | 14 |  |
| 3.30.30.200 | 1 | 3 |  |
| 3.30.30.50 | 1 | 1 | Translation initiation factor 2 beta, aIF2beta, N-terminal domain |
| 3.30.30.70 | 1 | 1 | Scorpion toxin-like |
| 3.30.30.90 | 3 | 14 | Polymerase Basic Protein 2, C-terminal domain |
| 3.30.310.180 | 1 | 8 |  |
| 3.30.360.60 | 1 | 7 |  |
| 3.30.370.20 | 1 | 4 |  |
| 3.30.380.10 | 4 | 233 | MS2 Viral Coat Protein |
| 3.30.387.10 | 1 | 109 | Viral Envelope Glycoprotein, domain 3 |
| 3.30.390.170 | 1 | 1 |  |
| 3.30.390.180 | 2 | 13 | RNA silencing suppressor P19 |
| 3.30.390.70 | 1 | 2 | Salmonella typhimurium protein |
| 3.30.40.140 | 1 | 8 |  |
| 3.30.40.20 | 2 | 10 | Chymotrypsin-like serine protease; domain 3 |
| 3.30.40.30 | 1 | 2 | YqaI domain |
| 3.30.420.330 | 2 | 69 | Influenza virus non-structural protein, effector domain |
| 3.30.420.410 | 1 | 23 | Arenaviral nucleoprotein, C-terminal domain |
| 3.30.420.560 | 1 | 2 |  |
| 3.30.428.20 | 2 | 24 | Rotavirus NSP2 fragment, C-terminal domain |
| 3.30.44.10 | 1 | 4 | Smk Toxin, beta chain |
| 3.30.450.250 | 1 | 1 |  |
| 3.30.450.340 | 1 | 3 |  |
| 3.30.450.390 | 2 | 2 |  |
| 3.30.450.420 | 1 | 5 |  |
| 3.30.460.60 | 1 | 9 | Poxvirus poly(A) polymerase, nucleotidyltransferase domain |
| 3.30.46.10 | 1 | 54 | Glycine N-methyltransferase, chain A, domain 1 |
| 3.30.470.140 | 1 | 6 |  |
| 3.30.470.40 | 1 | 1 |  |
| 3.30.500.30 | 3 | 6 |  |
| 3.30.500.50 | 1 | 2 |  |
| 3.30.530.60 | 1 | 27 |  |
| 3.30.56.50 | 1 | 1 | Putative DNA-binding domain, N-terminal subdomain of bacterial translation initiation factor IF2 |
| 3.30.60.110 | 1 | 4 |  |
| 3.30.60.190 | 1 | 1 |  |
| 3.30.60.280 | 1 | 15 |  |
| 3.30.67.10 | 3 | 109 | Viral Envelope Glycoprotein, domain 2 |
| 3.30.67.20 | 1 | 12 | Rubella membrane glycoprotein E1, domain 2 |
| 3.30.67.30 | 1 | 7 |  |
| 3.30.70.1370 | 1 | 2 | HD domain like |
| 3.30.70.1390 | 1 | 3 | ROC domain from the Parkinson's disease-associated leucine-rich repeat kinase 2 |
| 3.30.70.1600 | 1 | 71 |  |
| 3.30.70.1610 | 1 | 8 |  |
| 3.30.70.1690 | 1 | 41 |  |
| 3.30.70.1750 | 1 | 1 | Uncharacterised protein PF11491, DUF3213 |
| 3.30.70.1830 | 2 | 6 |  |
| 3.30.70.1840 | 2 | 58 |  |
| 3.30.70.2050 | 1 | 2 |  |
| 3.30.70.2060 | 1 | 2 |  |
| 3.30.70.2070 | 1 | 7 | VP9 protein domain |
| 3.30.70.2130 | 1 | 1 | Metalloenzyme domain |
| 3.30.70.2320 | 1 | 4 |  |
| 3.30.70.2370 | 1 | 1 |  |
| 3.30.70.2430 | 1 | 3 |  |
| 3.30.70.2440 | 1 | 23 |  |
| 3.30.70.2480 | 1 | 11 |  |
| 3.30.70.2640 | 1 | 16 | Arenavirus RNA polymerase |
| 3.30.70.2730 | 1 | 4 |  |
| 3.30.70.2770 | 1 | 1 |  |
| 3.30.70.2840 | 2 | 36 | Flavivirus RNA-directed RNA polymerase, thumb domain |
| 3.30.70.2850 | 1 | 2 |  |
| 3.30.70.2870 | 1 | 2 | Mastadenovirus E4 ORF3 |
| 3.30.70.2910 | 1 | 4 |  |
| 3.30.70.2940 | 1 | 1 |  |
| 3.30.70.3060 | 1 | 2 |  |
| 3.30.70.3110 | 1 | 4 |  |
| 3.30.70.3220 | 1 | 7 |  |
| 3.30.70.3490 | 1 | 11 |  |
| 3.30.70.3510 | 1 | 1 |  |
| 3.30.70.3530 | 1 | 1 | GCM motif |
| 3.30.70.3540 | 1 | 6 | Nsp8 replicase, head domain |
| 3.30.70.3590 | 1 | 2 |  |
| 3.30.70.3600 | 1 | 2 |  |
| 3.30.70.390 | 3 | 69 | Epstein Barr virus nuclear antigen-1, DNA-binding domain |
| 3.30.70.440 | 2 | 3 | Killer toxin KP6 alpha-subunit |
| 3.30.720.170 | 1 | 1 | Perilipin, alpha-beta domain |
| 3.30.720.180 | 1 | 1 |  |
| 3.30.720.190 | 1 | 21 |  |
| 3.30.720.60 | 1 | 6 |  |
| 3.30.730.30 | 1 | 1 | YaiA protein |
| 3.30.750.130 | 1 | 6 |  |
| 3.30.750.170 | 1 | 1 |  |
| 3.30.750.220 | 1 | 23 |  |
| 3.40.1000.70 | 1 | 1 | PknH-like extracellular domain |
| 3.40.1010.30 | 1 | 2 |  |
| 3.40.1170.50 | 1 | 1 |  |
| 3.40.1280.30 | 2 | 9 |  |
| 3.40.1310.10 | 2 | 14 |  |
| 3.40.1350.100 | 2 | 2 |  |
| 3.40.1350.110 | 1 | 2 |  |
| 3.40.1350.120 | 1 | 4 |  |
| 3.40.1350.140 | 1 | 1 | MepB-like |
| 3.40.1350.60 | 2 | 5 |  |
| 3.40.140.100 | 1 | 2 | Ubiquitin-like modifier-activating enzyme ATG7 C-terminal domain |
| 3.40.140.120 | 1 | 3 |  |
| 3.40.140.70 | 1 | 2 | Ubiquitin-like modifier-activating enzyme ATG7 N-terminal domain |
| 3.40.140.80 | 1 | 3 |  |
| 3.40.1580.30 | 1 | 6 | Domain of unknown function (DUF5066) |
| 3.40.1600.10 | 1 | 2 | PH1570-like |
| 3.40.1620.30 | 1 | 1 | ERCC4, Mus81-Eme1 complex, nuclease domain, subdomain 1 |
| 3.40.1620.80 | 1 | 1 | Big defensin, N-terminal domain |
| 3.40.1680.10 | 1 | 2 | yp_829618.1 domain like |
| 3.40.1760.20 | 1 | 2 |  |
| 3.40.1820.20 | 1 | 2 |  |
| 3.40.190.200 | 1 | 1 |  |
| 3.40.190.220 | 1 | 12 |  |
| 3.40.20.20 | 1 | 4 |  |
| 3.40.210.30 | 1 | 3 | Dam replacing family, catalytic PD-(D/E)XK domain |
| 3.40.220.20 | 1 | 13 | Nsp3, SUD-M subdomain |
| 3.40.220.30 | 1 | 6 | Nsp3, SUD-N subdomain |
| 3.40.250.20 | 1 | 1 |  |
| 3.40.30.150 | 1 | 2 | Coronavirus polyprotein cleavage domain |
| 3.40.30.160 | 1 | 7 | Collagenase ColT, N-terminal domain |
| 3.40.30.170 | 1 | 1 |  |
| 3.40.30.70 | 1 | 4 |  |
| 3.40.366.30 | 2 | 10 | 50S ribosomal protein L16 arginine hydroxylase; Chain A, Domain 2 |
| 3.40.390.70 | 1 | 2 |  |
| 3.40.390.80 | 1 | 2 | Peptidase M60, enhancin-like domain 2 |
| 3.40.50.10110 | 1 | 3 | DNA polymerase III subunit chi |
| 3.40.50.10220 | 1 | 3 | DNA polymerase III, psi subunit |
| 3.40.50.10280 | 1 | 6 | Methylene-tetrahydromethanopterin dehydrogenase, N-terminal domain |
| 3.40.50.10670 | 1 | 2 | af2093 domain |
| 3.40.50.10760 | 2 | 3 | Reovirus core |
| 3.40.50.10800 | 5 | 78 | NadA-like |
| 3.40.50.10870 | 1 | 6 | Glycosyl hydrolase family 3 |
| 3.40.50.11020 | 1 | 1 | Replicase polyprotein, nucleic acid-binding domain |
| 3.40.50.11130 | 1 | 27 | Glycoprotein VP7, domain 1 |
| 3.40.50.11230 | 5 | 15 |  |
| 3.40.50.11240 | 1 | 16 | Ethanolamine ammonia-lyase light chain (EutC) |
| 3.40.50.11300 | 1 | 2 |  |
| 3.40.50.11460 | 2 | 4 |  |
| 3.40.50.11480 | 1 | 95 |  |
| 3.40.50.11490 | 1 | 2 |  |
| 3.40.50.11500 | 1 | 2 |  |
| 3.40.50.11530 | 1 | 1 |  |
| 3.40.50.11580 | 3 | 27 |  |
| 3.40.50.11680 | 1 | 10 | Poxvirus mRNA capping enzyme, small subunit |
| 3.40.50.11790 | 1 | 1 |  |
| 3.40.50.11950 | 1 | 20 |  |
| 3.40.50.11960 | 1 | 1 |  |
| 3.40.50.11970 | 1 | 4 |  |
| 3.40.50.11980 | 2 | 20 |  |
| 3.40.50.11990 | 1 | 3 | RNA polymerase II accessory factor, Cdc73 C-terminal domain |
| 3.40.50.12020 | 1 | 12 | Uncharacterised protein family UPF0261, NN domain |
| 3.40.50.12030 | 1 | 10 | Uncharacterised protein family UPF0261, NC domain |
| 3.40.50.12050 | 1 | 4 |  |
| 3.40.50.12060 | 1 | 5 |  |
| 3.40.50.12080 | 1 | 4 |  |
| 3.40.50.12090 | 2 | 9 |  |
| 3.40.50.12100 | 2 | 79 | Stimulator of interferon genes protein |
| 3.40.50.12110 | 1 | 8 |  |
| 3.40.50.12120 | 1 | 1 | POC1 chaperone |
| 3.40.50.12140 | 1 | 1 | Domain of unknown function DUF4159 |
| 3.40.50.12150 | 1 | 32 |  |
| 3.40.50.12160 | 1 | 2 | Methylthiotransferase, N-terminal domain |
| 3.40.50.12170 | 1 | 2 | Uncharacterised protein PF07075, DUF1343 |
| 3.40.50.12180 | 1 | 2 |  |
| 3.40.50.12190 | 1 | 4 |  |
| 3.40.50.12230 | 2 | 26 |  |
| 3.40.50.12280 | 2 | 20 |  |
| 3.40.50.12430 | 1 | 2 |  |
| 3.40.50.12440 | 1 | 3 |  |
| 3.40.50.12550 | 1 | 19 | Ubiquitin-activating enzyme E1, inactive adenylation domain, subdomain 2 |
| 3.40.50.12600 | 1 | 5 |  |
| 3.40.50.12620 | 1 | 1 |  |
| 3.40.50.12640 | 1 | 20 | Phosphopantoate/pantothenate synthetase |
| 3.40.50.12650 | 2 | 314 |  |
| 3.40.50.12660 | 1 | 1 |  |
| 3.40.50.12670 | 1 | 2 |  |
| 3.40.50.12690 | 1 | 3 |  |
| 3.40.50.12700 | 1 | 3 |  |
| 3.40.50.12710 | 1 | 2 |  |
| 3.40.50.12760 | 1 | 4 |  |
| 3.40.50.12820 | 1 | 14 |  |
| 3.40.50.1780 | 2 | 11 |  |
| 3.40.5.100 | 1 | 14 |  |
| 3.40.5.30 | 1 | 2 | (Trans)glycosidases - domain 2 |
| 3.40.5.90 | 2 | 40 | CDGSH iron-sulfur domain, mitoNEET-type |
| 3.40.630.120 | 1 | 8 |  |
| 3.40.630.180 | 1 | 2 |  |
| 3.40.91.60 | 1 | 8 |  |
| 3.40.91.80 | 3 | 13 |  |
| 3.40.970.30 | 1 | 2 | yp_829618.1 like domains |
| 3.40.970.40 | 2 | 2 | fibrinogen binding protein from staphylococcus aureus domain like |
| 3.55.50.20 | 1 | 3 |  |
| 3.55.60.10 | 2 | 3 | Reovirus components |
| 3.90.1150.110 | 1 | 5 |  |
| 3.90.1150.20 | 1 | 10 | Transcription regulator MotA, C-terminal domain |
| 3.90.1150.90 | 1 | 1 |  |
| 3.90.1160.10 | 1 | 3 | Baseplate structural protein gp11, finger domain |
| 3.90.1310.20 | 1 | 7 |  |
| 3.90.1320.10 | 1 | 5 | Outer-capsid protein sigma 3, large lobe |
| 3.90.1370.10 | 1 | 3 | Protein mu-1, chain B, domain 1 |
| 3.90.1400.10 | 2 | 24 | Rotavirus NSP2 fragment, N-terminal domain |
| 3.90.1470.10 | 1 | 4 | thrh gene product, domain 2 |
| 3.90.1470.20 | 1 | 2 |  |
| 3.90.148.10 | 2 | 8 | Adenovirus DNA-binding, C-terminal domain superfamily/Adenovirus DNA-binding, zinc binding domain |
| 3.90.1620.10 | 1 | 26 | adenovirus 2 penton base, domain 2 |
| 3.90.1630.10 | 1 | 5 | Outer-capsid protein sigma 3, small lobe |
| 3.90.1720.60 | 1 | 2 |  |
| 3.90.1730.10 | 1 | 30 | Infectious bursal virus vp1 polymerase domain |
| 3.90.1810.10 | 2 | 3 | Reovirus components |
| 3.90.1830.10 | 1 | 2 | Inner capsid protein lambda-1 |
| 3.90.1840.10 | 1 | 2 | Major capsid protein |
| 3.90.1850.10 | 1 | 6 | RNA-directed RNA polymerase lambda-3 |
| 3.90.241.10 | 1 | 3 | Foki Restriction Endonuclease, Chain A, domain 1 |
| 3.90.249.10 | 1 | 19 | Hexon Major Viral Coat Protein, domain 3 |
| 3.90.380.20 | 1 | 19 | Herpesvirus glycoprotein H, domain D-II |
| 3.90.39.10 | 1 | 19 | Hexon Major Viral Coat Protein, domain 2 |
| 3.90.430.10 | 1 | 1 | Copper fist DNA-binding domain |
| 3.90.450.1 | 1 | 7 | Minor Coat Protein; Domain 2 |
| 3.90.550.60 | 1 | 4 |  |
| 3.90.55.10 | 1 | 18 | Dimethylsulfoxide Reductase, domain 3 |
| 3.90.640.80 | 1 | 1 |  |
| 3.90.660.60 | 1 | 12 |  |
| 3.90.70.100 | 1 | 5 |  |
| 3.90.70.110 | 1 | 12 | Alphavirus nsP2 protease domain |
| 3.90.70.150 | 1 | 1 | Helper component proteinase |
| 3.90.70.160 | 1 | 1 |  |
| 3.90.70.60 | 1 | 1 | Porcine arterivirus-type cysteine proteinase alpha domain |
| 3.90.70.70 | 1 | 1 | Arterivirus papain-like cysteine protease beta domain |
| 3.90.75.10 | 1 | 18 | Homing Intron 3 (I-ppo) Encoded Endonuclease; Chain A |
| 3.90.870.50 | 1 | 1 |  |
| 3.90.930.20 | 1 | 17 | Small outer capsid protein Soc |
|  |  |  |  |
